# Valuable Remedies

**Published:** 1877-06

**Authors:** 


### *VALUABLE REMEDIES.*

Almost every physician seems to have his "hobby." One will incorporate some preparation of *iron* in almost every prescription he writes. Another has such confidence in the virtues of *quinine*, that he employs it almost indiscriminately. Our preceptor and friend, Prof. H., surgeon in Bellevue Hospital, makes free use of hot water, and certainly with the most favorable results. We have, therefore, the highest authority for our belief in the power of certain remedies. Quinine is recognized by all physicians as a "specific" in intermittent fevers. Our experience, particularly during the last ten years, has taught us to believe that there are two other remedies that have fairly earned the title of specifics, since the most favorable results have always seemed to follow their use. One of these is "Van Deren's remedy" for "summer complaint of children," which we have given in thousands of the severest cases with the very best results. The remedy is contained in little sugar granules that any child will take readily. The price is 50 cents a box. No family where there are children, in city or country, should be without this remedy for a day during the summer months. It has saved thousands of lives.

Our other "specific" is "Nelaton's Suppository" for piles and constipation. Constipation is a most dangerous complaint if of long duration, as it is certain to be followed with serious and frequently fatal diseases. There is no complaint that a physician is so often called on to treat. These are now the very cases we desire, because we have found the treatment invariably satisfactory and the cures permanent. We wish that physicians and their patients could be induced to lay aside their prejudices against these remedies—simply because they are "advertised medicines"—long enough to give them a fair trial. We were once very unwise in this respect and caused our confiding patients much unnecessary suffering. The price of "Nelaton's Suppository" is 50 cents a box. All druggists should keep them. If our readers are not able to find them, they may send the price of either or both of the above remedies to Hall's Journal of Health, New York, and they will be sent promptly by mail post paid. One dollar will purchase both remedies and pay the postage to any point in the United States or Canada.

